# Evaluation of the Adhesive Properties of the Cornea by Means of Optical Coherence Tomography in Patients with Meibomian Gland Dysfunction and Lacrimal Tear Deficiency

**DOI:** 10.1371/journal.pone.0115762

**Published:** 2014-12-23

**Authors:** Pietro Emanuele Napoli, Franco Coronella, Giovanni Maria Satta, Maria Silvana Galantuomo, Maurizio Fossarello

**Affiliations:** Department of Surgical Sciences, Eye Clinic, University of Cagliari, Cagliari, Italy; School of Optometry and Ophthalmology and Eye Hospital, Wenzhou Medical University, Wenzhou, Zhejiang, China., China

## Abstract

**Objective:**

The aim was to determine the influence of meibomian gland dysfunction (MGD) and aqueous tear deficiency dry eye (ADDE) on the adhesive properties of the central cornea by means of optical coherence tomography (OCT), and to investigate the relationship between corneal adhesiveness and classical tear tests, as well as the reliability of results, in these lacrimal functional unit disorders.

**Design:**

Prospective, case-control study.

**Methods:**

Twenty-eight patients with MGD and 27 patients with ADDE were studied. A group of 32 healthy subjects of similar age and gender distribution served as a control group. The adhesive properties of the anterior corneal surface were measured by OCT, based on the retention time of adhesion marker above it, in all participants.

**Results:**

An excellent (≥5 minutes), borderline (within 3–5 minutes), fair (within 1–3 minutes) and poor (<1 minute) values of corneal adhesiveness were found, respectively, in 0%, 7.1%, 64.3% and 28.6% of MGD, in 0%, 7.4%, 63% and 29.6% of ADDE, and in 31.3%, 65.6%, 3.1% and 0% of healthy patients. The differences in time of corneal adhesiveness between MGD and healthy patients, as well as between ADDE and healthy patients, were found to be statistically significant (*p*<0.001; p<0.001; respectively). Conversely, no statistical significant differences between MGD and ADDE were found (*p* = 0.952). Data analysis revealed a statistically significant correlation between corneal adhesiveness and clinical tests of dry eye, as well as an excellent degree of inter-rater reliability and reproducibility for OCT measurements (*p*<0.001).

**Conclusion:**

ADDE and MGD share similar abnormalities on OCT imaging. Decreased adhesive properties of the anterior cornea were identified as a common feature of MGD and ADDE. This simple OCT approach may provide new clues into the mechanism and evaluation of dry eye syndrome.

## Introduction

Study of the precorneal tear film (PTF) and of the ocular surface has enjoyed renewed interest due to evidence that dry eye syndrome is a common ophthalmic condition which adversely impacts the quality of life of those who suffer from it. [1], [2]

One important trend in research has been the development of methods to characterize the lacrimal functional unit. [3]–[7] The measurement of quantitative and qualitative parameters of the tear film and of the ocular surface offers an objective approach to monitor the effect of dry eye disease on the conjunctival and corneal epithelia, as well as the results of proposed treatments. [8]

Since the PTF plays an essential role in quality of vision and in the health of the eye, the anterior surface of the cornea requires unique properties of adhesiveness to maintain a stable tear film. In particular, the mucus layer overlying the corneal epithelial cells normally promotes chemical interactions that retain the aqueous layer above it. These *attractive or adhesive* forces allow the molecules of tears to resist gravitational forces, especially on the central cornea where tear film drainage in human eyes is dominated by gravity. [9]

Optical coherence tomography (OCT) is a real-time instrument that has been applied to obtain detailed cross-sectional images of anterior tissues of the eye and to noninvasively assess human tear film behavior. [10]–[14] Recently, we described a novel OCT technique to measure the adhesive properties of the anterior corneal surface *in vivo*. [15] Potentially, this method may permit a new, more rapid, less invasive, and more reliable evaluation of tear system.

In the present work, our OCT technique was applied to quantify the changes over time of the PTF after instillation of an artificial tear (AT) in humans. For its good muco-adhesive capacities for human corneal epithelial cells, the sodium carboxymethylcellulose AT was used as adhesion marker. [16]

We assumed that the residence time of the adhesion marker on the central surface of a vertically oriented cornea, away from the menisci, might reflect the overall result of gravitational forces, blinking movements and adhesive properties of the anterior corneal surface. With this in mind, under the same conditions of gravity and normal blinking, OCT imaging was performed to measure the differences in time of the corneal adhesiveness for adhesion marker between meibomian gland dysfunction (MGD), aqueous tear deficiency dry eye (ADDE), and healthy patients. Moreover, we investigated the correlation between traditional tear tests and corneal adhesiveness, as well as inter-rater repeatability and reproducibility of our technique of OCT imaging for each group of patients.

## Methods

This study was approved by the Office of Research ethics of the University of Cagliari Hospital and was conducted in accordance with the tenets of the Declaration of Helsinki. Written informed consent was received from all the enrolled patients. The design of the present study was prospective.

Twenty-eight patients with MGD (53.17±15.8 years [mean ± standard deviation]; 78.6% female) and 27 patients with ADDE (54.22±17.21 years; 81.5% female) were studied. A group of 32 healthy subjects (52.37±16.57 years; 75% female) of similar age and gender distribution served as a control group.

Subjects with different external ocular diseases occurred in the previous 6 months, any evidence of abnormal blinking or lid abnormality, topical or systemic medication, history of contact lenses wear, history of eye surgery or systemic diseases, were excluded from the study.

Adult patients presenting with complaints of ocular irritation were evaluated by three examiners (PEN, FC, GMS) at the Eye Clinic (University of Cagliari, Italy). Clinical and instrumental exams were performed from July 2012 to May 2014.

On the day before OCT imaging, all subjects underwent a complete ophthalmological investigation including clinical history, and a series of clinical tests for dry eye performed in the following order: [17] McMonnies questionnaire, fluorescent break-up time (FBUT), fluorescein staining of the cornea and conjunctiva graded according to the Oxford system, Schirmer I test, and a slit lamp examination of the lid margins and meibomian glands. [8] Based on the results of these tests, patients were classified into one of three groups based on the following inclusion criteria:

*Healthy*: Subjects with no history of use of eyedrops, no symptoms of ocular irritation, FBUT≥10 seconds, Oxford scheme ≤1 or panel A, and a Schirmer I test score more than 5 mm were considered as healthy.

*Meibomian gland disease*: It was considered to be present when patient exhibited Schirmer I test>5 mm and all of the signs/findings: [18]

- Significant subjective symptoms (McMonnies questionnaire score >10, including the positive score obtained by questions about symptoms n.5 and n.6).
- Anatomic abnormalities around the meibomian gland orifices (presence of one or more of the following is positive):
  - irregularity of the lid margin;
  - vascular engorgement;
  - anterior or posterior displacement of the mucocutaneous junction.
- Obstruction of the meibomian glands (presence of both is considered positive):
  - decreased meibum expression by moderate digital pressure;
  - obstructive findings of the gland orifices by slit lamp biomicroscopy (pouting, plugging, or ridge).

*Aqueous tear deficiency dry eye*: If the subject (with no signs of MGD) exhibited all of the following characteristics: significant subjective symptoms (McMonnies questionnaire score >10, including the positive score obtained by questions about symptoms n.5 and n.6), a FBUT <10 seconds, a significant vital staining of the ocular surface (Oxford scheme ≥2 or panel B) and a Schirmer I test ≤5 mm.

### FBUT and fluorescein ocular surface staining

A 5 µl sample of 2% liquid fluorescein was applied to the bulbar conjunctiva with a micropipette. After instillation, a yellow filter (Kodak Wratten no. 12) was used to enhance contrast when assessing FBUT (within 10–30 sec) and staining of the ocular surface (within 1–2 min) with a biomicroscope (×10 objective, under blue-light illumination). [8] The appearance of the first dry spot at the center of the cornea was timed using a chronometer. Three evaluations of FBUT were conducted, and the mean value was taken for data analysis. [19] The extent of the corneal surface area stained was graded according to the Oxford system as follows: 1 = panel A, 2 = panel B, 3 = panel C, 4 = panel D, 5 = panel E, 6 = panel>E.

### Schirmer Test

After a 15-min rest, [8] without previously instilling anesthetic drops, Schirmer paper test strips (Alcon Laboratories, Fort Worth, TX) were placed over the lid margin at the junction of the lateral and middle thirds of the lower eyelid for 5 minutes. The millimeters of strip wetting were measured and recorded.

### Experimental procedure

All examinations were conducted in the same conditions of temperature (21.23±0.47°C), humidity (40±5.72%) and time of the day (between 3 PM to 5 PM) in a dimly lit consulting room. The temperature (within a range of 15–25°C) and humidity (within a range of 30%–50%) in the small consulting room where the study was conducted were controlled by central air conditioning and two humidifiers.

### Corneal Adhesiveness: Physical principles

**Figure 1:**
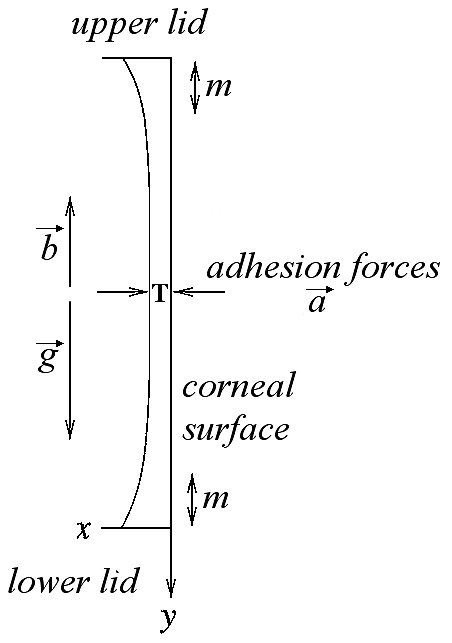
Schematic diagram of coordinate system (x, y) used for tear film behavior model. At baseline (pre-instillation, at time t = 0), the *central tear film thickness* (T) has a value T_0_≠0. After instillation of the adhesion marker (i.e., the artificial tear), at time t = 1, T has a value T_1_>T_0_, and is detected by OCT as a double-band structure (DB) above the epithelium of the cornea (see also Fig. 2). The *adhesive* forces between the adhesion marker and the anterior surface of the central cornea, away from the menisci (*m*), allow the molecules of the tear film to resist gravitational forces (*g*), especially on the central cornea where tear film drainage in human eyes is dominated by gravity. In the comparison between different patients, and under the same conditions of *g* and blinking movements (*b*), the residence time of AT on the epithelial surface clearly differ according to the characteristics of the individual ocular surface, and is assumed to be an index of the *corneal adhesiveness* (see text).

Considering a two-dimensional tear film in a Cartesian coordinates system (x, y) (a schematic diagram of coordinates system is shown in Fig. 1), since the radius of curvature of cornea is so much larger than the tear film, corneal surface is assumed to be a vertically oriented plate [9] positioned at y = 0.

**Figure 2:**
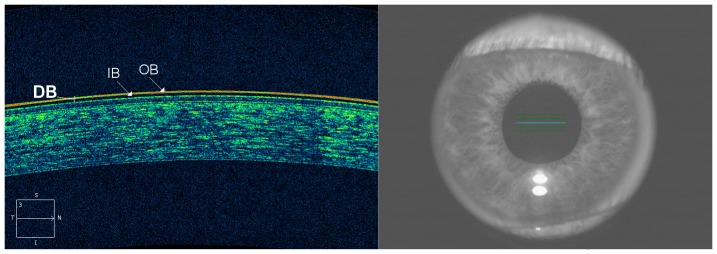
OCT image of the tear film and the central cornea (left), and simultaneous infrared image of the eye (right). Patients were asked to stare at a central target in the OCT. All OCT images of the ocular surface were acquired on the horizontal axis passing across the corneal apex. The instilled artificial tear (i.e., the adhesion marker) is detected by OCT as a two-layered structure localized onto the epithelial surface of the cornea, consisting of an outer band (OB) of high reflectivity and an inner band (IB) of low reflectivity. The retention time of the double-band structure (DB) onto the epithelial surface of the cornea was considered an index of the adhesive properties of the corneal surface (see text).

The *central tear film thickness* (T) at baseline (pre-instillation, at time t = 0) has a value T_0_≠0. After instillation of AT (i.e., the adhesion marker), at time t = 1, T has a value T_1_>T_0_. Unlike T_0_, T_1_ is detected by OCT as a double-band structure (DB) above the epithelium of the cornea (as described in Fig. 2).

Gravitational forces (*g*) tend to pull the molecules of tears down the ocular surface, causing the thinning of T. Conversely, blinking movements (*b*) have an important role in the partial reconstruction of T. Although *g* and *b* are vertical forces, adhesive forces (*a*) between tear film and corneal surface, which tend to hold them together, are horizontally oriented.

In the *comparison among different ocular surfaces* for T behavior after AT instillation (e.g., dry eye patient and healthy subject), we assume for each eye that *g* and *b* are similar, as well as chemical and physical properties of each calibrated drop of AT (35 µl, sodium carboxymethylcellulose 0.5%). In this way, the weight (w) of each drop is clearly the same for all subjects. Moreover, under the same conditions of temperature and humidity (as described above), the influence of the air above the tear film is assumed to be not significant in determining differences. Again, we assume to be constant the influence of circadian rhythm and of room brightness (as described above).

For all these reasons, the different behavior of T among patients (after AT instillation) is assumed to be mainly due to the *adhesive properties* of the anterior corneal surface. Under these circumstances, the residence time of AT on the epithelial surface clearly differ according to the conditions of the individual ocular surface and represents an index of the *corneal adhesiveness*.

### Corneal Adhesiveness: OCT measurements

In all patients, OCT scans were performed by using Cirrus HD-OCT 4000 (Carl Zeiss Meditec Inc., Dublin, California, USA). This Fourier-domain OCT system operates at 840 nm, and its acquisition speed is 27,000 A-scans per second. The axial and transverse resolutions are approximately 5 and 15 µm, respectively.

The Anterior Segment 5 Line Raster scanning protocol, which was used to obtain the cross-sectional images of the ocular surface, is composed of 5 equally separated cross-sectional anterior segment images covering an area of 3 mm (length) by 1 mm (width). Each line is composed of 4096 A-scans and each 5 Line Raster scan was taken approximately 0.75 seconds.

Patients were asked to stare at a central target in the OCT. One eye of each subject was randomly selected and imaged for session. The patient was asked to blink normally during the examination period and before each scan. Scans were acquired on the horizontal axis passing across the corneal apex. OCT imaging was performed at baseline and at four serial time-points after artificial tear instillation: immediately (within 30 seconds), at the 1^st^, at the 3^rd^ and at the 5^th^ minute. Particularly, thirty-five micro-liters of artificial tears (0.5% carboxymethylcellulose, Optive UD, preservative-free; Allergan, Inc., CA) were delivered using a pipette into the lower fornix of the study eye.

**Figure 3:**
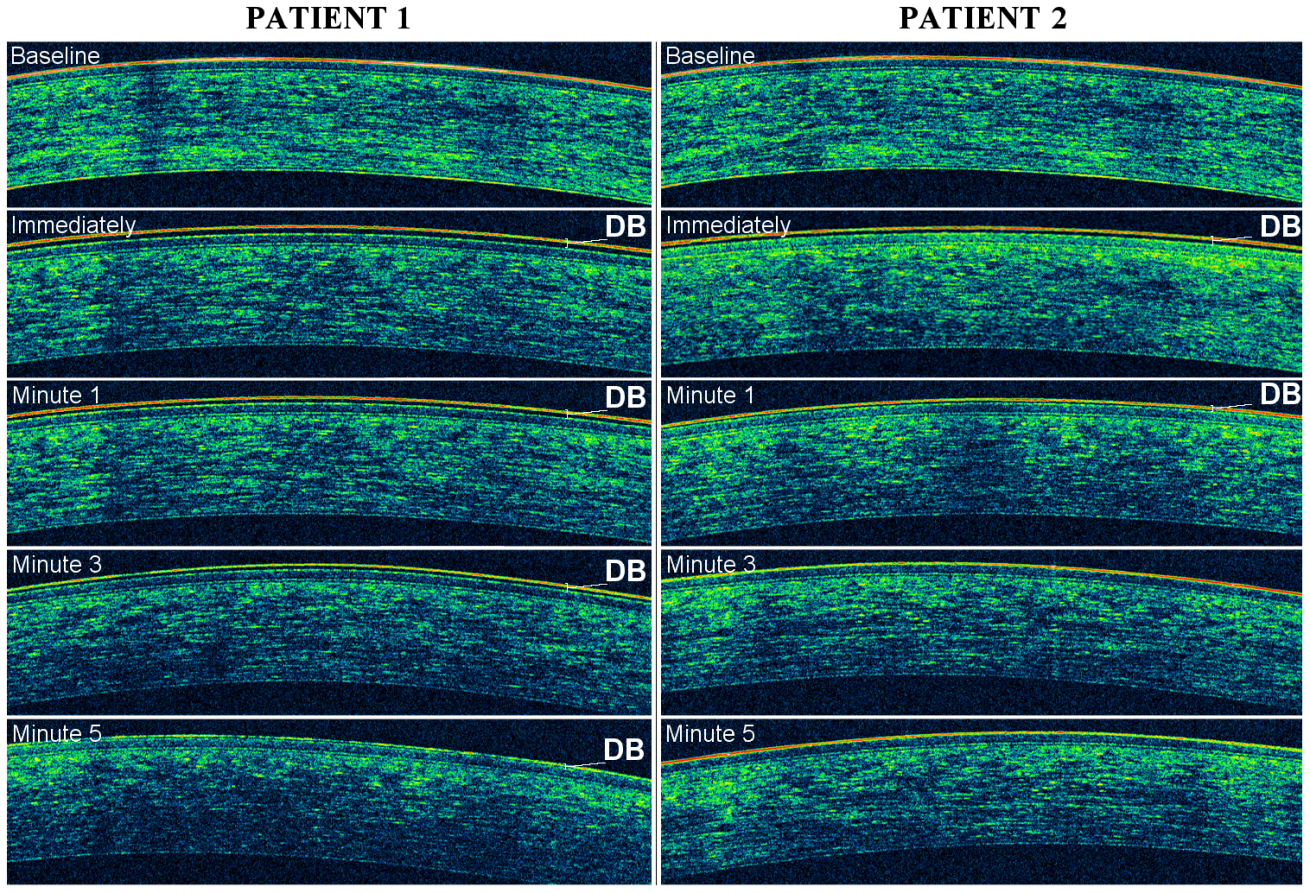
An example of excellent (patient 1) and fair (patient 2) grade of corneal adhesiveness. OCT images from patients 1 and 2 were obtained at baseline and at four serial time-points after artificial tear (i.e., the adhesion marker) instillation: immediately (within 30 seconds), at the 1^st^, at the 3^rd^ and at the 5^th^ minute. Changes of tear film behavior were noted during OCT imaging in both patients. The corneal adhesiveness was measured based on the residence time of the double line (DB), i.e. the velocity of progressive depletion (thinning) of the adhesion marker.

The evaluation of the *adhesive properties* of the anterior corneal surface was based on the dynamic behavior of adhesion marker (i.e., the instilled artificial tear) above it. Basically, the artificial tear is detected by OCT as a two-layered structure localized onto the epithelial surface of the cornea (Fig. 2), consisting of an outer band of high reflectivity and an inner band of low reflectivity. The retention time of the double-band structure onto the epithelial surface of the cornea, i.e. the velocity of its progressive depletion (thinning), was considered an index of the adhesive properties of the corneal surface (Figs. 2 and 3). With this in mind, we have graded the corneal adhesiveness into four levels: poor (between 0 and 1 minutes), fair (between 1 and 3 minutes), borderline (between 3 and 5 minutes) and excellent (at least for 5 minutes).

OCT measurements were performed on subsequent two days by two different examiners masked to the study in order to verify the reproducibility and inter-rater reliability of the results. To make an unbiased comparison between scans, best efforts were made by the operators to acquire the highest-quality images.

### Statistical analysis

Data analysis was carried out using Statistical Package for Social Science (SPSS version 21.0).

A sample size calculation was performed to determine the minimum ***N*** to identify a significant difference in corneal adhesiveness between groups. With an accepted two-sided statistical significance threshold of 0.05 and a β risk of 0.20 (i.e., 80% statistical power), and taking into account a 1∶1 group ratio, approximately 23 subjects were needed in each group. With this sampling scheme, it is possible to find statistically significant differences in corneal adhesiveness of 0.5 grades (score) or 0.5 minutes (time), showing that this technique is sufficiently precise for use in group-studies of corneal adhesiveness with moderate numbers of subjects. The common standard deviation was assumed to be 0.60 based on previous unpublished measurents in our Eye Clinic.

For analysis of corneal-adhesiveness *time*, the class interval arithmetic mean was obtained considering the mid-value for each category: 0.5, 2, 4, 6 (minutes) for poor, fair, borderline, excellent corneal-adhesiveness *grade*, respectively

OCT measurements obtained on the second day were used only for reproducibility analysis. Specific statistical tests are described as they are encountered in the article. P-values less than 0.05 were considered significant.

## Results

No significant differences in age and gender were found among groups (Kruskal-Wallis statistic = 0.122, *p* = 0.941; Kruskal-Wallis statistic = 0.36, *p* = 0.835; respectively). Descriptive statistics for diagnostic tests, as well as the statistically significant differences among the three studied groups are provided in Table 1, 2, 3 and 4.

**Table 1:** Patient Data.

	MGD Patients			ADDE Patients			Control Group Subjects		
	(n = 28)			(n = 27)			(n = 32)		
	Mean	± SD	Median (mode)	Mean	± SD	Median (mode)	Mean	±SD	Median (mode)
**McMonnies** (values)	16.4†	5.15	16 (9*)	20.1†	2.48	20 (20)	4	3.33	4 (4)
**FBUT** (sec)	6.75	2.04	6.5 (9)	5.25	2.36	6 (6)	14.8	5.5	12.5 (10*)
**Schirmer** (mm)	11.35	6.89	11 (11)	2.33	1.52	2 (2)	16.2	6.7	15.5 (13)
**Vital Staining** (score)	1.14	0.52	1 (1)	1.81	0.78	2 (1)	0	0	0
**Corneal Adhesiveness** (score) on the 1^st^ day**	1.78	0.56	2 (2)	1.77	0.57	2 (2)	3.28	0.52	3 (3)
**Corneal Adhesiveness** (minutes)†† on the 1^st^ day	1.7	0.93	2 (2)	1.7	0.95	2 (2)	4.56	1.04	4 (4)

MGD  =  Meibomian Gland Disease; ADDE  =  aqueous tear deficiency dry eye.

† = Symptomatic patients: the participants responded positively to question 5 and 6.

* = Multiple modes exist. The smallest value is shown.

** = OCT imaging was repeated on subsequent two days in order to verify the repeatability of our method.

†† = The Class Interval Arithmetic Mean (± SD) was obtained considering the mid value for each category: 0.5, 2, 4, 6 (minutes) for poor, fair, borderline, excellent (the largest values was 7 minutes) corneal adhesiveness grade, respectively.

McMonnies  =  McMonnies Questionnaire values; FBUT  =  Fluorescein Tear Break-up Time;

Schirmer  =  Schirmer I test; Vital Staining  =  Fluorescein Staining of the cornea and conjunctiva graded according to the Oxford system: 0 = panel A (grade = 1), 1 = panel B (grade = 2), 2 = panel C (grade = 3), 3 = panel D (grade = 4), 4 = panel E (grade = 5), 5 = panel>E (grade = 6); Corneal Adhesiveness score: 1 = poor (between 0 and 1 minutes), 2 = fair (between 1 and 3 minutes), 3 = borderline (between 3 and 5 minutes), 4 = excellent (greater than 5 minutes).

**Table 2:** Statistical Differences in Corneal Adhesiveness between MGD (Meibomian Gland Disease), ADDE (aqueous tear deficiency dry eye), and Healthy Patients.

	Corneal Adhesiveness
**Differences between groups**	
MGD-Healthy	*Mann–Whitney U statistic* = 31.5; *p*<0.001
ADDE-Healthy	“ ” = 32; *p*<0.001
MGD-ADDE	“ ” = 375; *p* = 0.952

*p* = Statistical Significance (two-tailed statistical analysis).

**Table 3:** Correlation Coefficients between Corneal Adhesiveness and Tear Tests in MGD (Meibomian Gland Disease), ADDE (Aqueous Tear Deficiency Dry Eye), and Healthy Patients.

Group of patients		Tear tests	Corneal Adhesiveness
	**MGD**	McMonnies, values	*Spearman's rho test* = − 0.470; *p* = 0.012
		FBUT, s	“ ” = 0.747; *p*<0.001
		Vital Staining, score	“ ” = −0.694; *p*<0.001
		Schirmer, mm	“ ” = 0.009, *p* = 0.963
	**ADDE**	McMonnies, values	*Spearman's rho test* = −0.519; *p* = 0.005
		FBUT, s	“ ” = 0.854; *p*<0.001
		Vital Staining, score	“ ” = −0.462; *p* = 0.015
		Schirmer, mm	“ ” = 0.646; p<0.001
	**Healthy**	McMonnies, values	*N/A* *
		FBUT, s	*Spearman's rho test* = 0.713; *p*<0.001
		Vital Staining, score	*N/A* *
		Schirmer, mm	*Spearman's rho test* = 0.532; *p* = 0.002

*p*  =  Statistical Significance (two-tailed statistical analysis).

* N/A  =  since healthy participants were selected with *constant* values of ocular discomfort ( =  no symptoms) and vital staining ( =  no ocular surface damage), correlation analysis was *not applicable* for these two parameters (i.e., McMonnies questionnaire score and Oxford scheme score, respectively).

McMonnies, values  =  McMonnies Questionnaire values; FBUT, s  =  Fluorescein Tear Break-up Time seconds; Vital Staining, score  =  Fluorescein Staining of the cornea and conjunctiva graded according to the Oxford system; Schirmer, mm  =  Schirmer I test score (millimeters).

**Table 4:** Intraclass Correlation for Corneal Adhesiveness Measurements in MGD (Meibomian Gland Disease), ADDE (Aqueous Tear Deficiency Dry Eye), and healthy Patients.

Group of patients		Intraclass Correlation Coefficient (ICC)^a^	
	**MGD**	Single measures	*ICC* = 0.881^b^
			95% CI (0.761, 0.943); *p*<0.001
		Average measures	*ICC* = 0.937^c^
			95% CI (0.864, 0.971); p<0.001
	**ADDE**	Single measures	*ICC* = 0.881^b^
			95% CI (0.757, 0.944); p<0.001
		Average measures	*ICC* = 0.937^c^
			95% CI (0.862, 0.971); p<0.001
	**Healthy**	Single measures	*ICC* = 0.891^b^
			95% CI (0.789, 0.945); p<0.001
		Average measures	*ICC* = 0.942^c^
			95% CI (0.882, 0.972); p<0.001

CI = 95% Confidential Interval (lower bound, upper bound).

*p* = Statistical Significance.

Two-way mixed effects model where people effects are random and measures effects are fixed.

a Type A intraclass correlation coefficients using an absolute agreement definition.

b The estimator is the same, whether the interaction effect is present or not.

c This estimate is computed assuming the interaction effect is absent, because it is not estimable otherwise.

An excellent, borderline, fair and poor value of corneal adhesiveness were found, respectively, in 0%, 7.1%, 64.3% and 28.6% of MGD patients, in 0%, 7.4%, 63% and 29.6% of ADDE patients, and in 31.3%, 65.6%, 3.1% and 0% of healthy subjects. Particularly, the mean time of corneal adhesiveness was 1.78±0.56 minutes in MGD patients, 1.77±0.57 minutes in ADDE patients, and 4.56±1.04 minutes in healthy subjects.

The differences in time of corneal adhesiveness between MGD and healthy patients, as well as between ADDE and healthy patients, were found to be statistically significant (Mann–Whitney *U* statistic = 31.5; *p*<0.001; Mann–Whitney *U* statistic = 32; *p*<0.001; respectively, Table 2). Conversely, no statistical significant differences between MGD and ADDE were found (Mann–Whitney *U* statistic = 375; *p* = 0.952).

**Figure 4:**
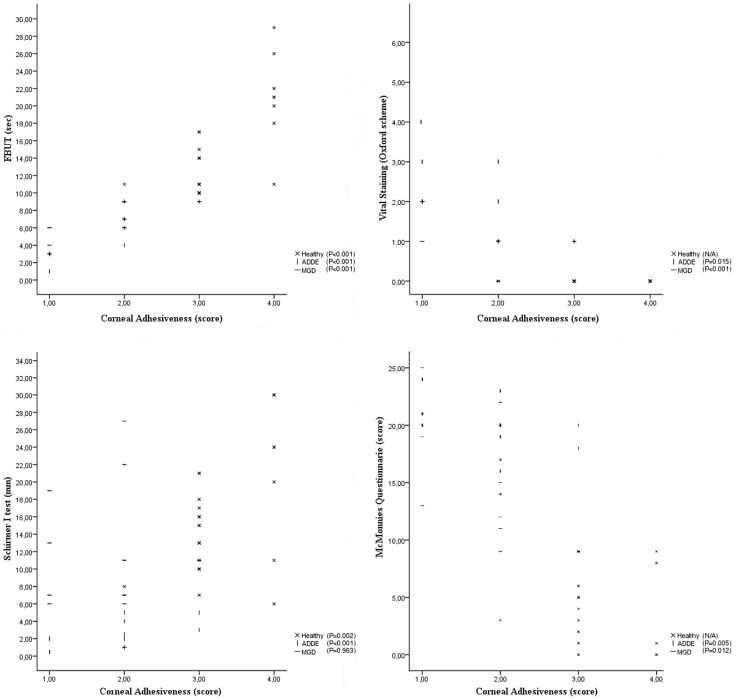
Correlation between corneal adhesiveness and traditional tear tests for each group of participants. The values of corneal adhesiveness were plotted on the X-axis as follows: 1 =  poor, 2 =  fair, 3 =  borderline, 4 =  excellent. Vital staining is plotted as follows: 0 =  panel A (grade = 1), 1 = panel B (grade 2), 2 = panel C (grade 3), 3 = panel D (grade 4), 4 = panel E (grade 5), 5 = panel > E (grade 6). Statistically significant correlations were noted in MGD (meibomian gland disease), ADDE (aqueous tear deficiency dry eye), and healthy patients. Particularly, FBUT (fluorescent break-up time) correlated very well to the corneal adhesiveness in each group.

Correlation analysis revealed in MGD patients a statistically significant association between corneal adhesiveness and dry eye symptoms, FBUT, and vital staining (Fig. 4; Table 3). Nevertheless, no correlation was found between corneal adhesiveness and Schirmer I test score in MGD group (Spearman's rho test = 0.009, *p* = 0.963). A statistically significant correlation was also found between all tear tests and corneal adhesiveness in ADDE patients (Fig. 4; Table 3). Similarly, a good association was demonstrated in healthy subjects between corneal adhesiveness and FBUT (Spearman's rho test = 0.713, *p*<0.001) or Schirmer I test score (Spearman's rho test = 0.532, *p* = 0.002). However, since healthy participants were selected with *constant* values of ocular discomfort ( =  no symptoms) and vital staining ( =  no ocular surface damage), correlation analysis was not applicable for these two parameters (i.e., McMonnies questionnaire score and Oxford scheme score, respectively).

Reproducibility analysis demonstrated a statistically significant intraclass correlation (ICC) value for OCT measurements of corneal adhesiveness time (ICC = 0.891, *p*<0.001 in healthy group; and ICC = 0.881, *p*<0.001 in both ADDE and MGD group), indicating the excellent inter-rater reliability and reproducibility of the test in various conditions of the tear film/ocular surface (Table 4).

## Discussion

One of the major ways for identifying the overall status of dry eye is the assessment of tear film surface quality. [20] The stability of the tear film depends on chemical-physical characteristics of that film interacting with the conjunctival and corneal epithelium via the membrane-spanning mucins (i.e., MUC-16 and MUC-4). [21], [22] Moreover, the presence of microvilli enhances the tear film stability by increasing the surface area of the plasma membrane. [23]

Tear film instability is considered to be an important condition that characterizes and defines the complex syndrome of dry eye. [20] In some forms of dry eye, tear film instability may be the initiating event, unrelated to prior tear hyperosmolarity. [23] Thus, tear film instability can be caused by epithelial damage involving cell death by apoptosis, a loss of goblet cells, and disturbance of mucin expression. [23] Moreover, in all forms of dry eye, tear film instability exacerbates ocular surface hyperosmolarity and promotes the *vicious circle* of dry eye syndrome. [24]

Nonetheless, as previously reported, [8] the lacrimal tests in clinical use for tear stability evaluation and dry eye diagnosis have many limitations. In a group of patients showing no obvious features of dry eye, Goto et al. demonstrated that not all aspects of tear film stability can be investigated by standard tests. [25]

In the present work, the OCT signal created by the artificial tear onto the epithelial surface of the cornea, represented by a double-band structure of different reflectivity, was evaluated during its progressive decay with time. In a vertically oriented tear system, the attractive or adhesive forces, represented by the interaction between the artificial tear polymer and the anterior surface of the cornea, clearly play a crucial role in determining the residence time of AT on the central cornea.

Although the new technique of OCT imaging described in our research does not evaluate the time to initial breakup of the tear film following a blink, it may quantify *in vivo* the adhesive properties of the cornea, providing an index of tear film stability. In fact, the contact time of the adhesion marker on the central cornea is a direct measure of the chemical interactions between mucins or epithelium cells and polymer. Accordingly, the results of our study show a very strong correlation between the time-length of corneal adhesiveness and FBUT.

With our method, we observed a reduced time of the corneal adhesiveness in MGD (which is the most common cause of evaporative dry eye) and in ADDE patients, i.e. in the two main sub-types of dry eye, compared with healthy controls. Considering that there is clinical evidence linking MGD to ADDE, [26] our study shows for the first time that they share similar abnormalities in corneal adhesiveness. This finding suggests that dry eye patients, independent of the subtype of dry eye, have in common a diminished ability in retaining the tears in front of their central corneal surface and, consequently, a reduced protection for the epithelia with an increased ocular surface exposure.

Since the surface epithelial damage and disturbance of glycocalyx and globet cell mucins are all aspects associated with the exposed surface, [23] we hypothesize that reduced adhesive properties of the cornea may promote and exacerbate the tear film instability as part of a vicious circle of events. On the other hand, the results of our study may also suggest, in case of abnormality of the ocular surface, a decreased time of contact between the epithelium of the *central cornea* and toxic factors, such as the pro-inflammatory cytokines, proteolytic enzymes and cytotoxic agents, which are in increased concentrations in altered hyperosmotic tears of dry eye patients.

All these aspects may appear contradictory with regard to the promotion and prevention of corneal damage, but they may simply suggest a different role of the corneal adhesiveness in the natural *history* of dry eye disease. Consequently, we strongly believe that future research should prospectively investigate and clarify the relationship between the adhesive properties of the corneal surface and dry eye syndrome. In this sense, our technique of OCT imaging may represent a new approach to understanding and diagnosing abnormalities of the ocular surface.

Moreover, since the adhesion marker (AT) applied in the present work has been found to bind to and be retained by human corneal epithelial cells, [16] clearly the time of corneal adhesiveness also provides an indirect measure of the ocular surface *integrity*. Indeed, a statistically significant correlation between corneal adhesiveness and vital staining has been demonstrated in our study.

Reduced corneal adhesiveness is expected in patients with aqueous tear deficiency because of dehydration of mucin with loss of its polar properties, and an alteration of mucin distribution and glycosilation. [27], [28] Accordingly, our results show that the corneal adhesiveness diminishes as the Schirmer test score decreases. However, in the case of MGD patients, the adhesive properties of the cornea are also reduced in patients with Schirmer “normal” (>10 mm wetting) or “borderline” (between 5 and 10 mm wetting). This corroborates our previous assertion that the corneal adhesiveness, independent of the level of aqueous tear production, shows a strongly correlation with tear film stability.

Diagnosing the cause of symptoms of ocular discomfort often represents a difficult problem to solve in dry eye patients. In the present study, we have reported that reduced adhesive properties of the cornea are a common finding in patients with ocular irritation, whether they have MGD or ADDE. This suggests that an alteration in corneal adhesiveness may have a role in determining dry eye symptoms, also in case of normal aqueous production. Particularly, a reduced corneal adhesiveness might be one of the mechanisms influencing the poor quality of vision (e.g., “fluctuation in vision”) in dry eye patients and, together with ocular surface inflammation, [29] that leads to “excessive tearing” in those who experience severe ocular irritation.

Interestingly, the adhesive properties of the cornea, representing an individual mechanism promoting tear film stability (e.g., different from normal tear evaporation), may constitute a potential therapeutic *target* for topical or systemic medication and an objective parameter to detect and monitor the *effect* of dry eye treatment.

The new method described in the present work for lacrimal functional unit evaluation provides several advantages compared with tear tests in clinical use. Unlike FBUT, our OCT technique eliminates the problems associated with sodium fluorescein instillation, prolonged eye-opening, and poor inter-rater reliability or reproducibility of results. [30] In fact, the tear film is not destabilized by sodium fluorescein, [31] and the possibility for the patient to blink normally prevents the ocular surface irregularities induced by a prolonged eye-opening, thereby avoiding the false-negative and false-positive results that may occur in both invasive and non-invasive FBUT. [32], [33] Compared with fluorometric analysis of the pre-corneal retention time of sodium fluorescein, OCT measurements of the corneal adhesiveness are not affected by autofluorescence of the cornea, as well as by the penetration of sodium fluorescein through the cornea (i.e., the staining and pseudo-staining of ocular surface, which induce a bias in the measurements). [34]

There are some limitations in the present study. First, a larger sample of normal, MGD, and ADDE patients should be created to evaluate possible age- and gender-variations, as well as to detect smaller, statistically significant differences in corneal adhesiveness between groups (e.g., between MGD and ADDE). Second, in our research, patients having history of eye surgery or systemic diseases, topical or systemic medication, ocular surface disease in past six months, any evidence of abnormal blinking or lid abnormality, or history of contact lenses wear, were excluded. However, all these factors are often found in dry eye patients and might have a role in determining variations in the adhesive properties of the cornea.

Future studies should evaluate and compare the corneal adhesiveness for other artificial tears by OCTs with higher resolution, in order to ameliorate our understanding of the physiopathology of the ocular surface, and to predict efficacy of proposed topical lubricants and tear film enhancement agents.

Finally, in our study we have been able to quantify in vivo a new parameter of tear film stability by using a simple, minimally invasive new test. The new OCT technique described herein is a useful addition to the diagnostic armamentarium for abnormalities of tear film/ocular surface, and may lead to a new era in studying the dry eye syndrome. For all these reasons, we believe that the analysis of adhesive properties of the cornea by OCT may have clinical and research relevance in the understanding and management of lacrimal functional unit abnormalities.
